# ZNF-281 as the Potential Diagnostic Marker of Oral Squamous Cell Carcinoma

**DOI:** 10.3390/cancers13112661

**Published:** 2021-05-28

**Authors:** Anna Starzyńska, Bartosz Kamil Sobocki, Aleksandra Sejda, Monika Sakowicz-Burkiewicz, Olga Szot, Barbara Alicja Jereczek-Fossa

**Affiliations:** 1 Department of Oral Surgery, Medical University of Gdańsk, 7 Dębinki Street, 80-211 Gdańsk, Poland; olga.szot@gumed.edu.pl; 2 International Research Agenda 3P—Medicine Laboratory, Medical University of Gdańsk, 3A Sklodowskiej-Curie Street, 80-210 Gdansk, Poland; 3 Department of Pathomorphology, University of Warmia and Mazury, 18 Żołnierska Street, 10-561 Olsztyn, Poland; aleksandra.sejda@uwm.edu.pl; 4 Department of Molecular Medicine, Medical University of Gdańsk, 7 Dębinki Street, 80-211 Gdańsk, Poland; ssak@gumed.edu.pl; 5 Division of Radiotherapy, IEO European Institute of Oncology, IRCCS, 435 Ripamonti Street, 20-141 Milan, Italy; barbara.jereczek@ieo.it; 6 Department of Oncology and Hemato-Oncology, University of Milan, 7 Festa del Perdono Street, 20-112 Milan, Italy

**Keywords:** oral squamous cell carcinoma, ZNF-281, diagnosis

## Abstract

**Simple Summary:**

Zinc-finger-281’s (ZNF-281’s) influence on carcinogenesis, progression and metastasis (through epithelial–mesenchymal transition induction) and its association with poor prognosis have been reported in many cancers. However, while reviewing the literature, there has been no mention of ZNF-281’s activity in oral squamous cell carcinoma (OSCC). In this pilot study, we analyzed *ZNF-281′s* expression and the impact of various clinical factors on its levels in OSCC patients. We aimed to verify whether ZNF-281 could potentially become a marker that will enable early OSCC diagnosis and help in assessing its prognostic value, specifically in terms of overall survival. Our findings show that the ZNF-281 H-score and mRNA expression were significantly decreased in OSCC when compared to healthy tissue, proving that ZNF-281 could become an OSCC-specific marker. ZNF-281’s role in OSCC should be further investigated as it is a promising candidate for an OSCC marker.

**Abstract:**

ZNF-281 is a zinc finger factor which can lead to cancer progression and metastasis. Its up-regulation reported in many cancers was correlated with metastasis and worsened patients’ prognosis. This is the first study describing ZNF-281 in the context of OSCC. Oral tissue samples drawn from 66 OSCC patients and 36 control patients were collected to determine protein (using immunochemistry and the semi-quantitative H-score method) and mRNA expression levels (using the RT-qPCR reaction). Our aim was to assess the ZNF-281 expression level in OSCC and the control group. Moreover, we determined the impact of ZNF-281 on survival parameters and the association of diversified clinical parameters with *ZNF-281* expression. Clinical factors such as grade, AJCC stage and radiotherapy have an impact on the ZNF-281 H-score level, whereas AJCC stage and grade have an influence on *ZNF-281* mRNA expression. Our survival analysis indicated that the impact on overall survival is not statistically significant, and the prognostic potential of ZNF-281 is rather limited. Our findings show that both levels of the ZNF-281 H-score and mRNA are decreased in OSCC in comparison to normal tissue. Moreover, we estimated that the H-score can differentiate normal tissue from OSCC with a sensitivity of 97% and specificity of 93.7%.

## 1. Introduction

Oral squamous cell carcinoma (OSCC) is the most common oral cancer, presenting in 90% of cases [1]. Unfortunately, OSCC is diagnosed at advanced stages (III and IV), when it has already metastasized to the neck lymph nodes. The main risk factors leading to OSCC are chronic tobacco and alcohol abuse, which, in the case of occurring concurrently, have a synergic effect. [2]. Men are at a two times higher risk of being affected by OSCC than women [3]. HPV-16 infection is another OSCC factor and usually is the cause of OSCC in younger patients with no history of alcohol or cigarettes overuse. It usually localizes at the vermilion border of the lower lip, the floor of the mouth and lateral border of the tongue [4]. Treatment at the early stages of OSCC consists of surgical resection or radiotherapy. For highly advanced OSCC, the surgery should be followed by radiation or chemoradiation [5]. The 5-year survival of patients with stages I and II is 82%. However, in the case of neck lymph node metastasis, it drops to 52%. This strongly proves the importance of early diagnosis and urgency for finding biomarkers of OSCC.

**Figure 1:**
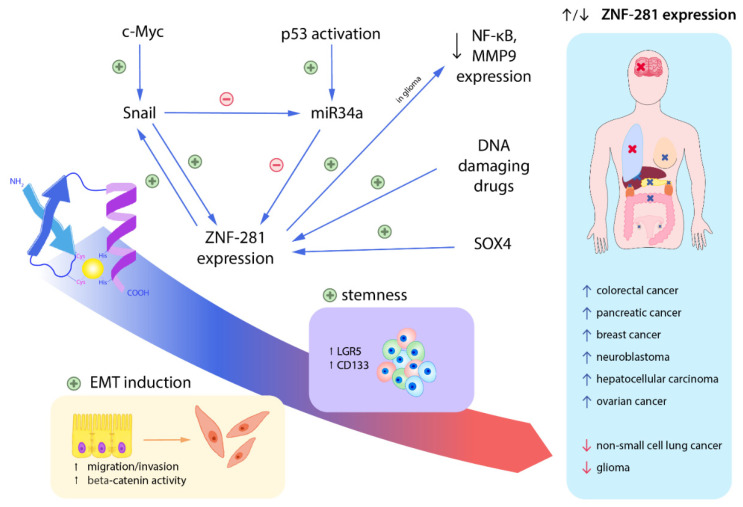
Regulation of *ZNF-281* expression and ZNF-281’s role in carcinogenesis. The illustration above shows the most important regulating factors of *ZNF-281* expression and outcomes of ZNF-281 stimulation. *SOX4* regulates cells’ differentiation and proliferation and induces EMT through ZNF-281. Another pathway stimulating EMT is initiated by Snail. ZNF-281 directly stimulates Snail, creating a positive feedback loop. Any DNA damage, including administering DNA-damaging drugs, will cause an increase in *ZNF-281* expression. p53 represses ZNF-281 and its inhibition is mediated by miR34a. ZNF-281 induces epithelial–mesenchymal transition (EMT) and keeps cells in the mesenchymal state, enabling cancerous cells to migrate and invade. Cells acquire a spindle shape, lose E-cadherin in the membrane and have β-catenin translocated from the membrane to the nucleus, resulting in weakening of cell junctions. *ZNF-281* induces expression of CD133 and LGR5 (prognostic stem cell markers) and is responsible for cells gaining pluripotency and maintaining their newly acquired stem cell traits. Up-regulated *ZNF-281* expression is proven to be correlated with progression and metastasis of the cancers listed above. In glioma, *ZNF-281* expression down-regulates NF-κB1 and MMP9 protein expression—both responsible for glioma cells’ invasiveness. In non-small cell lung cancer, *ZNF-281* gene expression suppresses carcinogenesis via promotion of apoptosis, inhibition of proliferation and tumor suppression. Therefore, ZNF-281’s down-regulation in glioma and NSCLC is correlated with its progression and metastasis.

ZNF-281 is a zinc finger factor that plays a major role in controlling cellular stemness expression and epithelial–mesenchymal transitions (EMT) by EMT induction and regulation of EMT-associated gene expression [6]. Research has shown that *ZNF-281* expression is positively correlated with DNA damage. DNA damage, induced by drugs, has caused an increase in *ZNF-281* expression in cell lines from diversified cancers [7]. Silencing *ZNF-281* expression results in delayed DNA repair. It has also been proven that the DNA repair mechanism is slowed down upon *ZNF-281′s* silencing due to its role in promoting DNA damage response genes—*XRCC2* (in breast cancer) and *XRCC4*—and stimulating nucleolin and cyclin B1 expression [6,7]. One can hypothesize that the decrease in *ZNF-281′s* expression or its dysfunction may lead to contributing to DNA instability and, finally, carcinogenesis. The influence of ZNF-281 on EMT and DNA repair mechanisms has been proven to be correlated with migration, invasion, stemness and metastasis. It is worth mentioning that transcription of *ZNF-281* is controlled by *SOX4*—an agent presented in many cancers and responsible for their key growth factors, developmental pathways and progression [6,7]. Up-regulation of *ZNF-281* gene expression was observed in colon cancer (protein expression), pancreatic cancer (protein and mRNA expression), breast cancer (protein expression), neuroblastoma (protein expression) and ovarian cancer (protein expression). High levels of ZNF-281 correlate with a higher metastatic potential and invasiveness and therefore result in a worse prognosis. However, in glioma and non-small cell lung cancer, ZNF-281 has an opposite effect on carcinogenesis. In glioma and NSCLC, ZNF-281’s down-regulation is associated with carcinogenesis, indicating that ZNF-281 might also have cancer-suppressing properties [8,9]. The Wnt/beta-catenin signaling pathway, promoting carcinogenesis and metastasis, was proven to be positively regulated by ZNF-281. Taking into consideration p53/miRNA-34a’s inhibitory effect on ZNF-281, it implies that any imbalance of the pathway will lead to cancer progression [6]. In the literature, the increase in *ZNF-281* expression is mostly correlated with gaining stem-like properties and induction of cancer progression and metastasis (Figure 1). However, study on glioma cells has shown that lnc-RNA-ZNF281 (found in the *ZNF-281* gene) might have a completely opposite effect on cancer progression and might inhibit glioma cell proliferation, migration and invasion, thus highlighting ZNF-281′s therapeutic value [8]. The fact that, in glioma and NSCLC (contrary to the majority of cancers), ZNF-281 is down-regulated raises a question concerning whether ZNF-281 always has oncogenic properties. The key to understanding ZNF-281’s role in cancer progression is to acknowledge that its expression is regulated by various cancer type-related miRNAs, phosphorylation and acetylation depending on the cell’s environment and protein co-factors, and its function may vary in different types of cancer [9].

No research has been conducted on ZNF-281’s activity in OSCC. Its ability to induce EMT and metastasis raises a question concerning whether *ZNF-281* could be one of the oncogenes responsible for oral squamous cell cancer progression and invasiveness. The aim of this study was to investigate the levels of *ZNF-281* expression in oral cancer and ZNF-281′s use in terms of becoming a new prognostic marker (mirroring OSCC stage and grade and delivering a more precise estimation of the survival rate) and diagnostic marker differentiating OSCC from normal tissue.

## 2. Results

### 2.1. ZNF-281 Expression and Its Connection with Cancer and Other Clinical Factors

**Figure 2:**
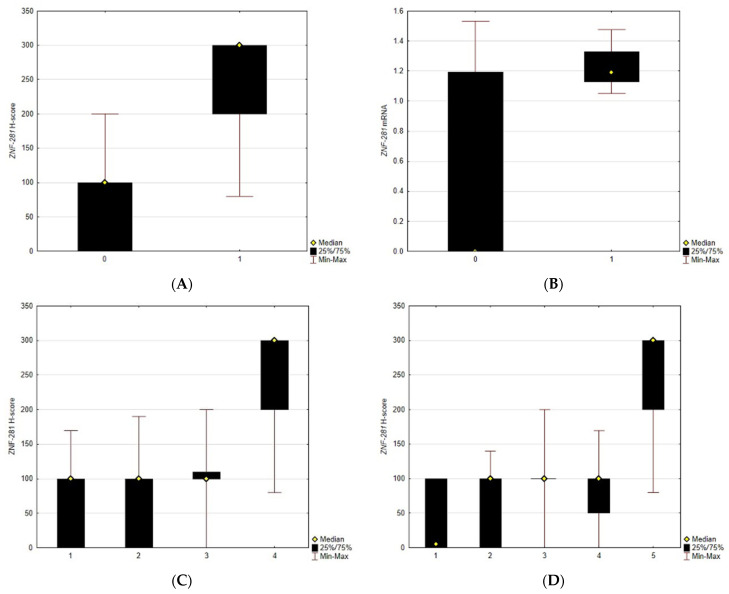

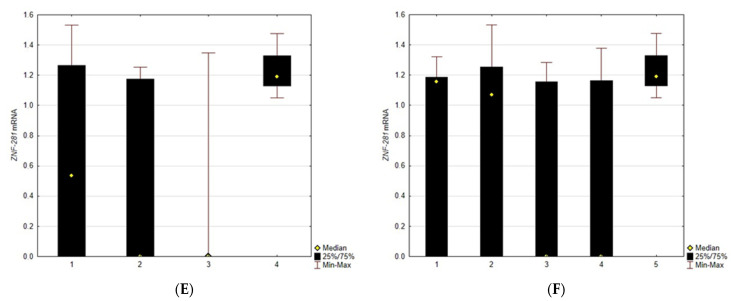
(**A**) Comparison of ZNF-281 H-score between OSCC and control, *p* < 0.001. (**B**) Comparison of *ZNF-281* mRNA transcript level between OSCC and control, *p* = 0.0011. (**C**) Comparison of ZNF-281 H-score in grades (1–3) and control. (**D**) Comparison of ZNF-281 H-score level in stages (1–4) and control. (**E**) Comparison of *ZNF-281* mRNA level in grades (1–3) and control. (**F**) Comparison of *ZNF-281* mRNA level in stages (1–4) and control.

Our analysis showed that sex, age, localization (Figure 2), arterial hypertension, diabetes mellitus, chemotherapy, surgical resection, alcohol abuse and cigarette smoking are not associated with ZNF-281 mRNA and protein expression levels. Only patients treated with radiotherapy have a significantly lower H-score (*p* = 0.006). All of the clinical parameters and their analyses are detailed in Table 1 and Table 2. 

Patients included in this study presented various localizations of OSCC—the most common localization was the floor of the mouth (36%), followed by the tongue (22%), lower gingivae (17%), cheek (13%), upper gingivae (10%) and palate (2%). In our group of 66 patients with OSCC, we observed 19 without *ZNF-281* expression and 45 patients with H-score > 50 (which we classified as positive). The maximum H-score was 200. The median H-score was 100 in OSCC and 300 in the controls. In the control group, the minimal H-score was 80, and the maximal H-score was 300. The expression level of mRNA (normalized with ACTB as a reference gene) was measured with RT-qCR in the group of 48 patients with OSCC. We observed 26 patients without *ZNF-281* mRNA expression. The mean of mRNA in OSCC patients was 0.562 (±SD 0.623), maximum was 1.531 and minimum was 0. In the control mRNA group, we observed zero patients without *ZNF-281* mRNA expression, and the mean was 1.207 (±SD 0.122), maximum was 1.477 and minimum was 1.05. We observed that the ZNF-281 H-score (*p* < 0.001) and *ZNF-281* mRNA (*p* = 0.0011) were significantly lower in OSCC than in the control group. A significantly higher H-score was observed in the control group in reference to grade 1 (*p* < 0.001), grade 2 (<0.001), stage 1 (*p* < 0.001), stage 2 (*p* < 0.001), stage3 (*p* = 0.028) and stage 4 (*p* < 0.001). A significantly higher mRNA expression level was found in the control group in reference to grade 2 (*p* = 0.034) and stage 4 (*p* = 0.015). Any other statistically significant differences between the control group and grades or stages, or amongst grades and stages, were not revealed. Distributions of the ZNF-281 H-score and mRNA results according to grades and stages, as well as the comparison with the control group, are depicted in Table S1 and Figure 2.

### 2.2. ZNF-281 as a Diagnostic Marker

**Figure 3:**
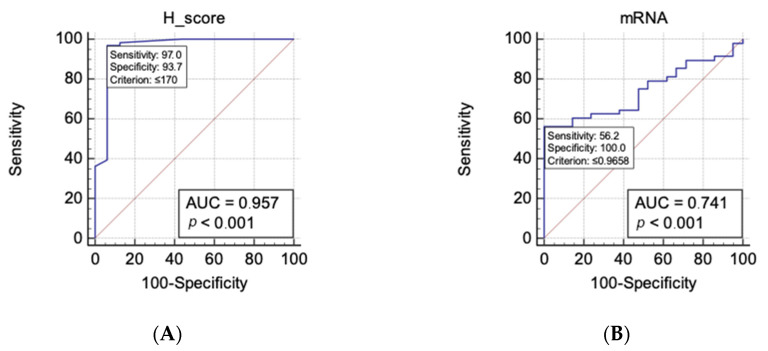
ROC curves for differentiation of OSCC from normal tissue and their accuracy using (**A**) H-score and (**B**) ZNF-281 mRNA transcript level normalized with ACTB gene.

Having confirmation that the ZNF-281 H-score level is significantly different in OSCC and the control group, we tried to set up the level of the H-score to be appropriate for differentiation of OSCC from the control group. Receiver operating characteristic curve (ROC) analysis (Figure 3) revealed that the area under curve was 0.957 (95% CI 0.888–0.989). Our analysis using the Youden index showed that the optimal cut-off for confirmation of OSCC was ≤170, with a sensitivity of 97% (95%CI 89.5–99.6) and specificity of 93.7% (95% CI 69.8–99.8). Analysis of the *ZNF-281* mRNA level was also conducted. The area under curve was 0.741 (95% CI 0.621–0.839). The optimal cut-off of the relative mRNA expression level for confirmation of OSCC was ≤0.966, with a sensitivity of 56.2% (95% CI 41.2–70.5) and specificity of 100% (95% CI 83.9–100).

### 2.3. Impact of ZNF-281 on Survival

**Figure 4:**
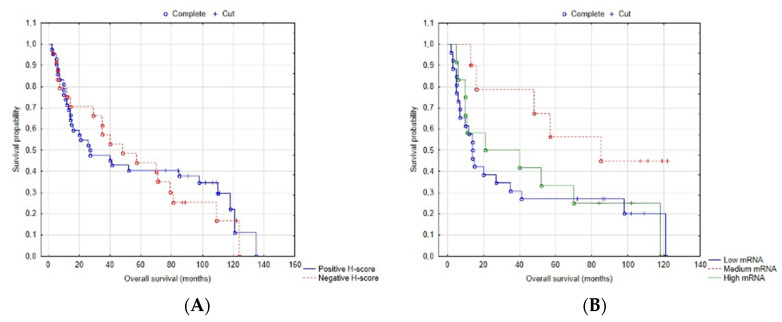
Overall survival analysis according to ZNF-281 level of: (**A**) H-score—patients with positive H-score (>50, *n* = 42) vs. negative H-score (<50, *n* = 24), log-rank *p* = 0.77; and (**B**) mRNA—patients were divided according to mean ± SD into three groups: low (<0.50, *n* = 26), medium (0.50 < x < 1.22, *n* = 12) and high (>1.22, *n* = 10), log-rank *p* = 0.55.

The number of deaths in this group was 47 (71.2%). The median overall survival (OS) was 37.5 (2–135) months, and 5-year disease-free survival was 43.9%. In order to determine the impact of the ZNF-281 H-score and mRNA relative expression level on overall survival, we analyzed our data using the Kaplan–Meier curve (Figure 4). During analysis according to the H-score, we divided the population (*n* = 66) into two groups: positive (>50) and negative (≤50) H-scores. Our analysis showed that there is no significant difference between high and low ZNF-281 H-scores in the overall survival result (log rank, *p* = 0.77). In analysis of the mRNA impact on OS, patients were divided according to mean ± SD into three groups, with low (<0.50, *n* = 26), medium (0.50 < x < 1.22, *n* = 12) and high (>1.22, *n* = 10) mRNA expression levels. Statistical differences amongst these three groups were not observed (log-rank, *p* = 0.55). Similarly, the multiple Cox regression model with adjustments for all analyzed clinical factors showed that the ZNF-281 H-score and mRNA relative expression are not appropriate predictors of OS. The univariate Cox regression model also confirmed the mRNA multiple model results (H-score: *p* = 0.774, mrNA = 0.148). Cox analysis was performed in the group of 66 patients (with H-score only) and repeated for 48 patients from this group (with H-score and mRNA analysis) separately. The multiple Cox regression model showed that in the group of 66 patients, the following clinical factors were not remarkable for OS: localization (*p* = 0.92), sex (*p* = 0.23), age (*p* = 0.15), grade (*p* = 0.3), stage (*p* = 0.8), pT (*p* = 0.72), diabetes (*p* = 0.52), hypertension (*p* = 0.68), surgery (*p* = 0.29), radiotherapy (*p* = 0.92), alcohol abuse (*p* = 0.96), whereas in the group of 48 patients, these were as follows: age (*p* = 0.15), sex (*p* = 0.23), localization (*p* = 0.92), pT (*p* = 0.63), pN (*p* = 0.8), grade (*p* = 0.55), stage (*p* = 0.47), radiotherapy (*p* = 0.99), surgery (*p* = 0.47), hypertension (*p* = 0.06), diabetes (*p* = 0.59), alcohol abuse (*p* = 0.3), smoking (*p* = 0.4). Significant predictors of hazard are detailed in Table 3 and Table 4. In the end, we also assessed that there is no correlation between H-score and mRNA relative expression levels of ZNF-281 and 5-year disease-free survival.

## 3. Discussion

This is the first description of *ZNF-281* gene expression in oral squamous cell carcinoma. First of all, in order to assess the impact of different clinical factors on ZNF-281 protein and mRNA expression levels, we conducted an analysis. As a result, it was revealed that only the presence of radiotherapy treatment has an impact on the ZNF-281 H-score. This could be explained as a side effect of radiotherapy, as it generally causes a decrease in protein levels.

We expected that ZNF-281 has a significant impact on OS, but our study did not confirm any statistically important impact of ZNF-281 on OS and 5-year DFS.

After the analysis of the impact on other clinical parameters, we reported that the ZNF-281 H-score was significantly decreased in OSCC. In our group of patients, we observed that the H-score was significantly higher in the control group than in G1 or G2. However, significant differences between the control and G3 or amongst grades were not observed. Analysis according to AJCC stage indicated that there is also a significant difference between all stages and the control group, but no difference amongst stages. The analysis of mRNA expression showed that it was significantly decreased in OSCC in comparison with the control group. In addition, mRNA was notably higher in the controls than in grade 2 and stage 4. Our data show that both the H-score and mRNA are not precise markers of clinical and pathological development of cancer. This could be associated with the fact that ZNF-281 has an impact on different mechanisms with opposite effects on cancer progression such as metastasis and DNA repair [7]. However, we confirmed a significant difference between OSCC and the control group in both the H-score level and mRNA expression. In order to evaluate and objectify the potential of ZNF-281 as a diagnostic marker, we found the optimal cut-off for the H-score (<170), with a sensitivity of 97% and specificity of 93.7%. In contrast, the cut-off for mRNA (≤0.966) has a specificity of 100%, but a sensitivity of 56.2%. This shows that mRNA has a limitation in sensitivity. However, the H-score is a potential biomarker for OSCC diagnosis and confirmation. 

Diagnostic biomarkers such as ZNF-281 can be really helpful during pathological diagnosis, especially in poorly differentiated OSCC and when tissue is collected after adjuvant radiotherapy, and we tried to assess the presence of a recurrence in tissue. Changes after radiotherapy can imitate cancer in normal tissue. This is the reason why markers such as ZNF-281 can support pathological diagnosis and make it more accurate.

The majority of cancer studies emphasized that *ZNF-281* gene up-regulation correlates with cancer presence, progression, metastasis and poor prognosis. These results were observed in pancreatic [10], breast [7], colon [6,7] and many other cancers. Basing on the literature, we expected up-regulation of OSCC in OSCC. However, in our study, both mRNA and protein expressions were down-regulated in OSCC. As it was mentioned in the introduction, ZNF-281 participates in epithelial–mesenchymal transition and thus has an impact on poor prognosis. However, it has also been proven that silencing the *ZNF-281* gene leads to perturbations in DNA repair and that DNA damage increases the expression of this molecule [7]. It is also commonly known that cancer cells show different types of mutations and aberrant expressions of genes involved in the DNA repair response, which lead to genome instability and carcinogenesis [11]. Based on our data, we suppose that DNA repair deficiency (because of a low level of ZNF-281) dominates over others and leads to cancer development in OSCC. In normal oral tissues, ZNF-281 may actively participate in DNA repair, when in cancer, decreasing levels of ZNF-281 may lead to instability and carcinogenesis. This may be the potential reason why we observe down-regulation of ZNF-281 in OSCC when there is up-regulation in the majority of cancers. However, we strongly recommend critical evaluation of this hypothesis. We found that down-regulation of *ZNF-281* gene expression also exists in glioma and non-small cell lung cancer (NSCLC). In glioma, it is confirmed that ZNF-281 reduces glioma stem-like cells’ invasive ability via down-regulation of NF-κB1 and MMP9 protein expression in vitro and in vivo [8]. We know that the NF-κB1 pathway has an essential role in cancer invasion; therefore, we strongly recommend focusing on this molecular pathway and its interactions with ZNF-281 in OSCC. In NSCLC, overexpression of the *ZNF-281* gene, resulting in promotion of cell apoptosis and inhibition of cell proliferation, exhibits tumor-suppressive properties. It is worth pointing out that this study in NSCLC shows that ZNF-281 down-regulates miR-221, which has an impact on the up-regulation of PTEN. This study also indicates that there are direct interactions of ZNF-281 and miR-221 with NF-κB1; therefore, we can conclude that NF-κB1 regulates the interaction between ZNF-281 and miR-221 in OSCC, but this hypothesis also needs further evaluation. These detailed pathways may also be involved in the down-regulation effect of ZNF-281 in OSCC [12]. As we mentioned before, *ZNF-281* expression is regulated by various cancer type-related miRNA expressions, phosphorylation and acetylation, which depends on the specific tissue’s microenvironment. This is the reason why in one type of cancer we observe the existence of up-regulation, whereas in others, the existence of down-regulation is noticed. The mechanisms which regulate the expression of *ZNF-281* in OSCC have still not been investigated, and further research in this topic is required.

In our group, we did not conduct statistical analysis investigating the interaction between HPV infection and ZNF-281 due to the lack of an adequate number of patients with an HPV diagnosis. HPV infection is also an OSCC risk factor, especially among younger patients. This virus encodes two oncoproteins: E6 and E7 [13]. E6 and E7 are the genes responsible for carcinogenesis. In this process, E6-associated protein inactivates p53—a tumor suppressor that is also a part of the signaling pathway inhibiting *ZNF-281’s* expression [14,15]. This is the reason why HPV infection could have an impact on ZNF-281, and further analysis of this correlation should be conducted.

This study is a pilot study of ZNF-281 as a marker in OSCC. The main limitation is the relatively low number of patients included in this study. However, we conducted rigorous statistical analysis and applied restricted inclusion criteria in order to reduce bias. Further research on a larger group of patients is necessary. The strengths of our study include the usage of unique clinical material, thorough clinical documentation, stringent criteria for patient selection, the study design and the statistical analysis. Our study suggests that ZNF-281 could be a potential new marker of OSCC. It also indicates strategic areas of possible research, and it represents the first analysis of ZNF-281 in OSCC.

## 4. Materials and Methods

### 4.1. Participants and Design of the Study

This study was approved by the local ethics committee of the Medical University of Gdańsk, Poland (NKBBN/373-705/2019). Retrospective analysis was conducted in the group of 66 white race patients (Figure S1) with oral squamous cell carcinoma hospitalized at the Maxillofacial Surgery Department in the University Clinical Centre in Gdańsk. The material was collected during surgical resection or the diagnostic biopsy procedure. Written consent was obtained from every patient. Clinical information was collected and consisted of age, sex, grade, TNM stage, location, presence of hypertension and diabetes, type of applied treatment (chemotherapy, radiotherapy, surgical resection), alcohol abuse, smoking cigarettes, overall survival (measured from diagnosis) and 5-year disease-free survival. Patients with recurrence or incomplete clinical data were excluded from our analysis. TNM stages were classified according to the 8th edition of the AJCC Cancer Staging Manual. Material was graded (G1–G4) and qualified to our analysis by an experienced pathologist. 

### 4.2. Immunohistochemistry

**Figure 5:**
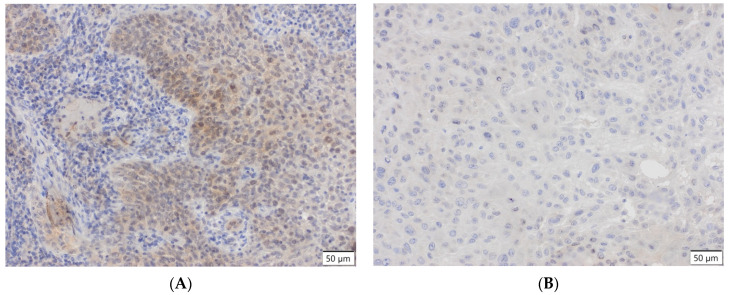
(**A**) ZNF-281-positive, 20×, scale bar 50 μm. (**B**) ZNF-281-negative, 20×, scale bar 50 μm.

All surgically removed specimens were fixed in 4% buffered formalin and routinely embedded in a low-melting paraffin. Paraffin blocks were used to construct tissue microarrays using a Manual Tissue Arrayer MTA 1 device (Beecher Instruments Inc, Sun Prairie, WI, USA). The hollow needle was used to extract tissue cores from donor blocks which were re-embedded into a single recipient block to form a microarray. Then, all blocks were cut into sections of 4 μm thickness on the microtome (Leica SM 2000, Nussloch, Germany). Before staining, the slides were incubated for 24 h at 37 °C, deparaffinized and rehydrated. For antigen retrieval, the heat-induced epitope retrieval method was used (PTLink, Dako, Glostrup, Denmark). For blocking, endogenous peroxidase slides were incubated in 3% H_2_O_2_ for 5 min. Manual staining procedure was conducted using the Dako EnVision Flex/HRP system (Glostrup, Denmark). Immunohistochemical staining was performed using rabbit Anti-ZNF-281 primary antibody (Sigma, HPA051228, 1:200, Saint Louis, MO, USA) (Figure 5).

### 4.3. H-Score Analysis

The microscope glass slides were analyzed under a light microscope. For each core, the intensity (weak, medium, strong) and percentage of positively stained cells were quantified. The semi-quantitative H-score coefficient was calculated using the following formula: percentage of weakly staining cells + percentage of moderately staining cells × 2 + percentage of strongly staining cells × 3. The final points range from 0 to 300. The H-score analysis was performed independently by two pathologists. If any differences between pathologists were revealed, a third specialist would review the score.

### 4.4. The Expression of mRNA

Isolation of total RNA from FFPE was conducted using the RNeasy FFPE Mini Kit by QIAGEN N (Qiagen GmbH, Hilden, Germany). V according to the manual user. The material for isolation was collected from 48 FFPE freshly cut into 8 to 10 5 μm-thick fragments. After the isolation, the amount of total RNA from FFPE was fluorometrically detected with Quant-iT kit (Thermo Fisher Scientific, Warszawa, Poland) according to the protocol and manual user. Then, the mRNA level of the *ZNF-281* gene was normalized with the reference gene *ACTB*, using the TaqMan probes during the RT-qPCR reaction. The RT-qPCR reaction was conducted with the use of TranScriba One step qPCR kit (A&A Biotechnology, Gdańsk, Poland) and Universal ProbeLibrary for Human (probe *#70* for *ZNF-281* mRNA) (Roche Diagnostics International Ltd., Rotkreutz, Switzerland) in a LigtCycler 480 II device (Roche Diagnostics International Ltd., Rotkreutz, Switzerland). The following specific primer for tested transcripts was used (*ZNF-281*: 5′-ggagaggacggcgttatttt-3′, (5′-gaggaggccccatactttt-3′)). For the reference gene *ACTB*, Universal ProbeLibrary Human *ACTB* Gene Assay (Roche Diagnostics GmbH, Mannheim, Germany) was used. Tested transcript levels were normalized to the *ACTB* transcript relative expression level.

### 4.5. Statistical Analysis

Analysis of using ZNF-281 as a marker was assessed with MedCalc software 19.6.1 (Ostend, Belgium). Every next statistical analysis was performed using STATISTICA 13.3 (StatSoft Inc., Tulsa, OK, USA). In our analysis, *p* < 0.05 was considered as statistically significant. Normal distribution of data was verified with the Shapiro–Wilk test. The impact of different clinical parameters on *ZNF-281* gene expression was analyzed with Mann–Whitney *U* test and Kruskal–Wallis one-way ANOVA test. To assess the appropriate cut-off for differentiation of OSCC from controls, the ROC curve and Youden index were analyzed. Survival analysis was evaluated with the Kaplan–Meier method and investigated for significance by the log-rank test. Moreover, univariate and multiple Cox regression models adjusted for all available clinical factors were constructed. The REMARK guidelines were used for the precise and objective description of ZNF-281 as a biomarker [16].

## 5. Conclusions

This is the first study that describes ZNF-281 in OSCC. Firstly, we investigated how clinical factors influence ZNF-281 levels. It has been proven that grade, AJCC stage and radiotherapy have an impact on the ZNF-281 H-score level, whereas AJCC stage and grade have an influence on *ZNF-281* mRNA expression. Secondly, we conducted survival analysis in reference to the ZNF-281 H-score and mRNA, but we did not receive any significant results. Thirdly, we compared levels of the ZNF-281 H-score and mRNA in OSCC and the control group. Statistically significant differences between these groups and the level of both mRNA and H-score results are the most promising and meaningful results of our study. Our analysis indicated that mRNA (≤0.966) could be an OSCC biomarker with a specificity of 100% and sensitivity of 56.2%. Moreover, the H-score (<170) is even more promising as a diagnostic marker with a sensitivity of 97% and specificity of 93.7%. This all shows that ZNF-281’s prognostic role is rather limited, but the diagnostic role in OSCC should be further investigated. 

## Figures and Tables

**Table 1:** Impact of different factors on ZNF-281 H-score (protein) level. Clinical characteristics of 66 patients with H-score.

Sex	Age	Hypertension	Diabetes	Radiotherapy	Chemotherapy	Alcohol Abuse	Cigarette Smoking	Surgical Resection	Grade	AJCC Stage	Classification	
											T	N
N (%)	Media (range)	0 = no, 1 = yes (%)							(1–3): N (%)	(1–4): N (%)	(1–4): N (%)	(0–3): N (%)
Female 20 (30.3)	60 (40–83)	0: 34 (51.5)	0: 57 (86.4)	0: 33 (50)	0: 62 (93.9)	0: 53 (80.3)	0: 19 (28.8)	0: 14 (21.2)	1: 29 (43.9)	1: 16 (24.2)	1: 16 (24.4)	0: 38 (57.6)
									2: 30 (45.5)	2: 13 (19.7)	2: 22 (33.3)	1: 9 (13.6)
Male 46 (69.7)		1: 32 (48.5)	1: 9 (13.6)	1: 33 (50)	1: 4 (6.1)	1: 13 (19.7)	1: 47 (71.2)	1: 52 (78.8)		3: 11 (16.7)	3: 13 (19.7)	2: 13 (19.7)
									3: 7 (10.6)	4: 26 (39.4)	4: 15 (22.7)	3: 6 (9)
Protein												
*p* = 0.903	*p* = 0.175	*p* = 0.19	*p* = 0.501	*p* = 0.006	*p* = 0.095	*p* = 0.765	*p* = 0.599	*p* = 0.234	*p* < 0.001	*p* < 0.001	*p* = 0.0934	*p* = 0.3119

For the group of 82 patients (66 with OSCC + 16 controls, H-score), the *p*-value was estimated to find any significant difference in ZNF-281 level according to Grade, AJCC Stage and T and N classifications with the Kruskal–Wallis one-way ANOVA test, whereas that according to other parameters was estimated with the Mann–Whitney *U* test.

**Table 2:** The impact of different factors on *ZNF-281* mRNA levels. Clinical characteristics of 48 patients with mRNA levels.

Sex	Age	Hypertension	Diabetes	Radiotherapy	Chemotherapy	Alcohol Abuse	Cigarette Smoking	Surgical Resection	Grade	AJCC Stage	Classification	
											T	N
N (%)	Media (range)	0 = no, 1 = yes (%)							(1–3): N (%)	(1–4): N (%)	(1–4): N (%)	(0–3): N (%)
Female 15 (31.25)	60 (40–83)	0: 27 (56.3)	0: 39 (81.3)	0: 26 (54.2)	0: 45 (93.8)	0: 38 (79.2)	0: 15 (31.3)	0: 13 (27.1)	1: 29 (43.9)	1: 16 (24.2)	1: 10 (20.8)	0: 25(52)
									2: 30 (45.5)	2: 13 (19.7)	2: 16 (33.3)	1: 6 (12.5)
Male 33 (68.75)		1: 21 (43.8)	1: 9 (18.8)	1: 22 (45.8)	1: 3 (6.3)	1: 10 (20.8)	1: 33 (68.8)	1: 36 (75.0)		3: 11 (16.7)	3: 13 (27)	2: 13 (27)
									3: 7 (10.6)	4: 26 (39.4)	4: 9 (18.8)	3: 4 (8.3)
mRNA												
*p* = 0.214	*p* = 0.075	*p* = 0.803	*p* = 0.614	*p* = 0.71	*p* = 0.926	*p* = 0.846	*p* = 0.322	*p* = 0.472	*p* = 0.0071	*p* = 0.0085	*p* = 0.145	*p* = 0.5295

For the group of 68 patients (48 with OSCC + 20 controls, mRNA), the *p*-value was estimated to find any significant difference in *ZNF-281* level according to Grade, AJCC Stage and T and N classifications with the Kruskal–Wallis one-way ANOVA test, whereas that according to other parameters was estimated with the Mann–Whitney *U* test.

**Table 3:** Multiple Cox regression model for group of 66 patients: significant predictors of hazard and non-significant ZNF-281 H-score. Analysis of impact on OS of ZNF-281 H-score with adjustments for all clinical factors.

Factor	CI	Std. Error	CI 95%	95% CI Upper	Cox Regression	Wald	*p*	Relative Risk
H-score	−0.00353	0.003894	−0.01116	0.00410	−0.90626	0.821306	0.364805	1.00974133
pN	0.89532	0.332906	0.24284	1.54780	2.68941	7.232919	0.007162	2.80803567
Chemotherapy	−2.89423	0.930870	−4.71870	−1.06976	−3.10916	9.666903	0.001878	0.135787
Smoking	1.07314	0.433994	0.22253	1.92376	2.47272	6.114325	0.013414	3.673476

**Table 4:** Multiple Cox regression model for the group of 48 patients: significant predictors of hazard. Analysis of impact on OS of both ZNF-281 H-score and mRNA with adjustments for all clinical factors.

Factor	CI	Std. Error	CI 95%	95% CI Upper	Cox Regression	Wald	*p*	Relative Risk
H-score	−0.00631	0.004497	−0.01513	0.00250	−1.40400	1.971206	0.160330	0.993732
mRNA	−0.28358	0.418067	−1.10297	0.53582	−0.67830	0.460095	0.497585	0.931273
Chemotherapy	−2.68493	1.143477	−4.92610	−0.44376	−2.34804	5.513285	0.018879	0.239034
Radiotherapy	1.05475	0.435139	0.20189	1.90761	2.42394	5.875467	0.015359	3.610641
Hypertension	−1.60461	0.608912	−2.79806	−0.41117	−2.63521	6.944341	0.008413	0.308258

## Data Availability

The data presented in this study are available on request from the corresponding author.

## Supplementary Materials

The following are available online at https://www.mdpi.com/article/10.3390/cancers13112661/s1, Figure S1: Description of patients groups in study and characterization of their qualification to analysis, Table S1: *p* value for multiple comparisons (two-sided) with control and stage (H-score), grade (H-score), stage (mRNA), grade (mRNA).
