# Supplementary material for: ZNF-281 as the Potential Diagnostic Marker of Oral Squamous Cell Carcinoma
Source: Cancers (Basel). 2021 May 28;13(11):2661. doi: 10.3390/cancers13112661 (PMC8197962; doi:10.3390/cancers13112661)
Supplement: Supplementary file 1 [file cancers-13-02661-s001.zip › cancers-1217018-supplementary.pdf]

cancers MDPI

Supplementary Material: ZNF-281 as the Potential Diagnostic Marker of Oral Squamous Cell Carcinoma

Anna Starzyńska, Bartosz Kamil Sobocki, Aleksandra Sejda, Monika Sakowicz-Burkiewicz, Olga Szot and Barbara Alicja Jereczek-Fossa

Table S1. *p* value for multiple comparisons (two-sided) with control and (**A**) stage (H-score).(**B**) grade (H-score). (**C**) stage (mRNA). (**D**) grade (mRNA).

	**Stage 1**	**Stage 2**	**Stage 3**	**Stage 4**	**Control**
Stage 1		1.000000	0.359205	1.000000	0.000000
Stage 2	1.000000		1.000000	1.000000	0.000225
Stage 3	0.359205	1.000000		1.000000	0.028413
Stage 4	1.000000	1.000000	1.000000		0.000031
Control	0.000000	0.000225	0.028413	0.000031	

(**A**)

	**Grade 1**	**Grade 2**	**Grade 3**	**Control**
Grade 1		1.000000	0.907800	0.000001
Grade 2	1.000000		1.000000	0.000004
Grade 3	0.907800	1.000000		0.116236
Control	0.000001	0.000004	0.116236	

(**B**)

	**Stage 1**	**Stage 2**	**Stage 3**	**Stage 4**	**Control**
Stage 1		1.000000	1.000000	1.000000	1.000000
Stage 2	1.000000		1.000000	1.000000	1.000000
Stage 3	1.000000	1.000000		1.000000	0.066743
Stage 4	1.000000	1.000000	1.000000		0.015029
Control	1.000000	1.000000	0.066743	0.015029	

(**C**)

	**Grade 1**	**Grade 2**	**Grade 3**	**Control**
Grade 1		1.000000	1.000000	0.149373
Grade 2	1.000000		1.000000	0.017676
Grade 3	1.000000	1.000000		0.115378
Control	0.149373	0.017676	0.115378	

(**D**)

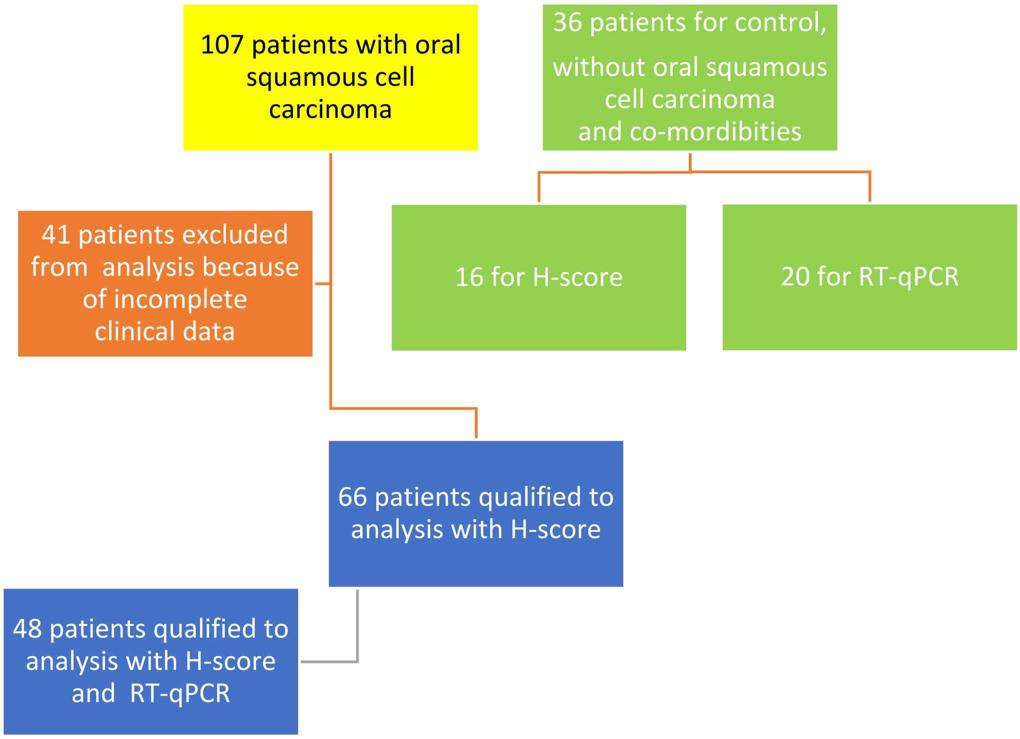

Figure S1. Description of patients groups in study and characterization of their qualification to analysis.
